# Comparison of Different Methods for Separation of Haploid Embryo Induced through Irradiated Pollen and Their Economic Analysis in Melon (*Cucumis melo* var. *inodorus*)

**DOI:** 10.1155/2013/529502

**Published:** 2013-05-29

**Authors:** Gökhan Baktemur, Hatıra Taşkın, Saadet Büyükalaca

**Affiliations:** ^1^Department of Biology, Faculty of Arts and Sciences, University of Osmaniye Korkut Ata, 80000 Osmaniye, Turkey; ^2^Department of Plant Production and Technologies, Faculty of Agricultural Sciences and Technologies, University of Niğde, 51240 Niğde, Turkey; ^3^Department of Horticulture, Faculty of Agriculture, University of Çukurova, 01330 Adana, Turkey

## Abstract

Irradiated pollen technique is the most successful haploidization technique within *Cucurbitaceae*. After harvesting of fruits pollinated with irradiated pollen, classical method called as “inspecting the seeds one by one” is used to find haploid embryos in the seeds. In this study, different methods were used to extract the embryos more easily, quickly, economically, and effectively. “Inspecting the seeds one by one” was used as control treatment. Other four methods tested were “sowing seeds direct nutrient media,” “inspecting seeds in the light source,” “floating seeds on liquid media,” and “floating seeds on liquid media after surface sterilization.” Y2 and Y3 melon genotypes selected from the third backcross population of Yuva were used as plant material. Results of this study show that there is no statistically significant difference among methods “inspecting the seeds one by one,” “sowing seeds direct CP nutrient media,” and “inspecting seeds in the light source,” although the average number of embryos per fruit is slightly different. No embryo production was obtained from liquid culture because of infection. When considered together with labor costs and time required for embryo rescue, the best methods were “sowing seeds directly in the CP nutrient media“ and ”inspecting seeds in the light source.”

## 1. Introduction

Melon (*Cucumis melo* L.), which belongs to the *Cucurbitaceae *family, is one of the important vegetables because of a rapid increase in its production and nutrient value. Melon is rich in terms of protein, minerals and vitamins, such as Vitamin A (500–4200 IU/100 g) and Vitamin K (130–330 mg/100 g) [1]. In recent years, it has become widespread to use melon in fruit salad and fruit juice as well as consuming it freshly. Moreover, it is used in other branches of the food industry (pastry, jam, ice-cream, and fruit yogurt). Its immature fruits can be used in making pickles and can be used in making soup in the Far East. Some melon species are also known to be used as ornamental plants. It is also common in perfumery and cosmetic industry such as in shampoos and perfumes [2]. Melon holds a great economic value in the world in terms of human nutrition and 28 million ton production in 1.3 million ha area annually. Turkey ranks second among the world's melon-producing countries with 103000 ha area and total production of 1.75 mt [3]. 

Obtaining homozygote pure lines through conventional breeding techniques in melon requires a long period such as 10–12 years, and 100% homozygous cannot provide because of open pollinations. Therefore, these techniques can conduct with more quick and effective *in vitro* techniques such as “haploidy techniques.” It is possible to get 100% homozygote pure lines in one year by obtaining plants that have haploid chromosome number and doubling their chromosome numbers with colchicine. Hence, homozygotation process can be shortened for 1 to 2 years. Toward 1990s, *in sitü* haploid embryo stimulation by pollination with gamma irradiated pollens and germination of these embryos in special nutrient media were studied, and this process was performed in cucumber [4, 5], melon [6–8], watermelon [9, 10], and squash [11]. Because the number of the plants obtained was adequate and regular in melon and cucumber, this method was started to be used in breeding program of melon and cucumber. The most common method to obtain haploid embryo is “inspecting the seeds one by one.” However, this method takes long time and needs laboratory staff. There are a limited number of studies on the use of different methods. In a study conducted for obtaining double haploid lines through irradiated pollen technique in cucumber, embryos in seeds were determined through X-ray [12, 13]. However, this method is not widely used because it requires special equipment as an X-ray source. Lotfi et al. [14] planted the seeds into the fluid culture, and the seeds germinated. In some of our previous studies, the seeds were examined in a light source; the ones which were detected to have embryo were separated and opened. 

The main objective of this study is to explore the time and labor cost of the four methods that are “sowing seeds into direct nutrient media,” “inspecting seeds in the light source,” “floating seeds on liquid media,” and “floating seeds on liquid media after surface sterilization” as an alternative to the method of “inspecting the seeds one by one,” to compare the time of the best practice and unit costs counted for each method, and to put forward the least-cost method.

## 2. Material and Methods

### 2.1. Material

Y2 and Y3, the third backcross (BC3) population of Yuva (local recurrent cultivar) selected from a melon breeding project conducted at the Department of Horticulture, Faculty of Agriculture, University of Çukurova, were used as plant materials in this study. Resistance melon genotypes to 0, 1, and 3 races of Fusarium wilt and Yuva melon genotypes were crossed to obtain BC3 population.

### 2.2. Methods

#### 2.2.1. Greenhouse Practices and Embryo Stimulation

**Figure 1:**
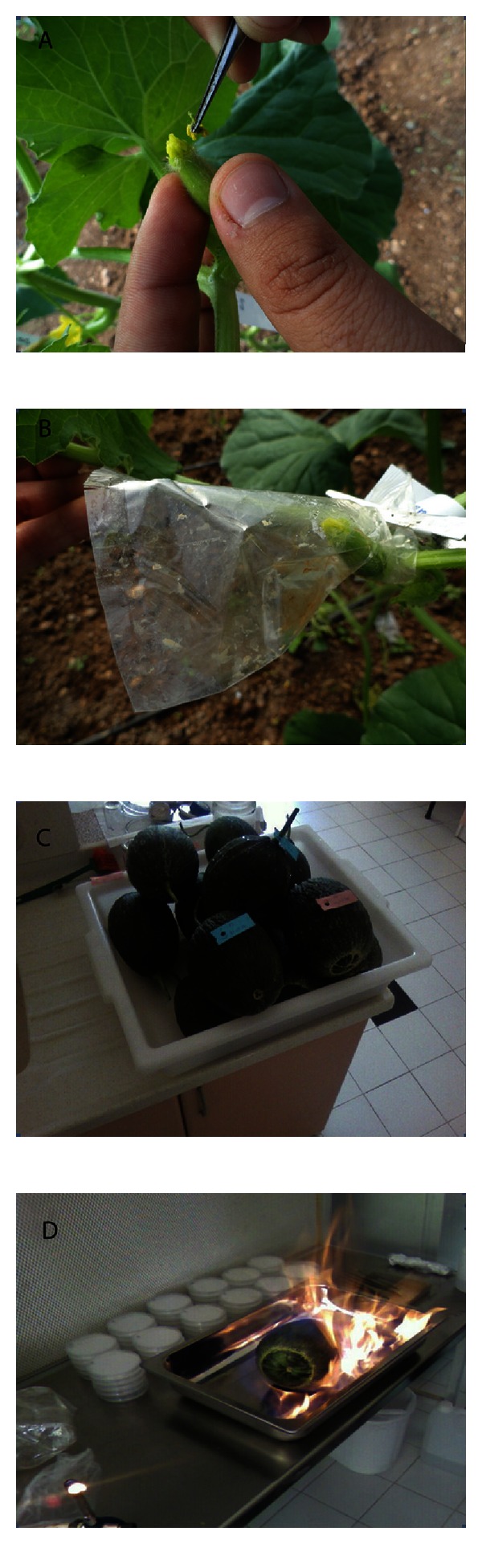
(A) Pollination of female flowers with irradiated male flowers, (B) closing of pollinated female flowers with cellophane bags to prevent pollen contamination, (C) harvested melon fruits, (D) disinfection of melon fruits with 96% ethyl alcohol through dry burning method.

This study was conducted in a 326 m^2^-plastic greenhouse belonging to the Department of Horticulture, Faculty of Agriculture, University of Çukurova. Seeds were sown plugs containing soil mixture (2 volumes peat : 1 volume perlite) in February. Seedlings were transferred to plastic greenhouse with double-row systems (50 × 50 × 100 cm space) in March. Throughout the growing period, normal horticultural cultivation practices were implemented. Pollination with irradiated pollen was performed between 16th April and 16th May, which was suitable for the spring breeding period. Male flowers were collected one day before anthesis (the stage when petals started to change their color from green to yellow). After they were separated from petals and partially sepals, they were put into glass petri dishes for irradiation. Female flowers were emasculated with pens and closed with cellophane bags to prevent open pollination on the same day. Irradiation was performed at the Department of Radiation Oncology, Faculty of Medicine, University of Çukurova, with 300 Gy gamma ray coming from Co^60^ [7]. Irradiated male flowers were waited in room temperature during the night. The next day, female flower was emasculated the day before being pollinated by irradiated male flowers. After pollination, flowers were closed with cellophane bags again to pollen contamination. In the forthcoming days, the state of pollination was checked, and cellophane bags were removed in the period when the ovary of the female flower began to swell and the stigma became dry (Figure 1). 

#### 2.2.2. Embryo Extraction

**Figure 2:**
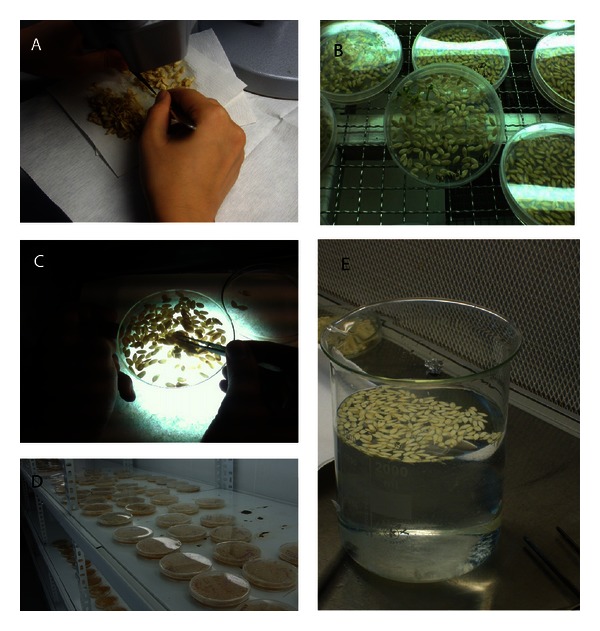
(A) Method 1. (B) Method 2. (C) Method 3. (D) Method 4. (E) Method 5.

Melon fruits were harvested between the dates of 11th May and 21st June, 21–25 days after pollination. After harvested fruits were washed with tap water and dried, they were placed in a sterile jar and disinfected with 96% ethyl alcohol through dry burning method (Figure 1). Then, the fruits were separated into two by lengthwise cutting, and the seeds inside were extracted and put into sterile petri dishes. Five different methods were tested for the embryo rescue (Figure 2).

*Method 1.* All the seeds were opened one by one under stereo binocular microscope and checked whether they contained embryo. Finding embryos were placed in glass culture tubes containing E20A nutrient medium [6, 12] with 8 g L^−1^ agar.

*Method 2.* Seeds without exposure to any treatment were directly sown into sterile plastic 5-cm-diameter petri dishes containing CP medium [15] with 30 g L^−1^ sucrose, 8 g L^−1^ agar, 0.08 mg L^−1^ Vitamin B12, 0.02 mg L^−1^ IAA and E20A medium with 8 g L^−1^ agar. The sown seeds were observed regularly for the first 20 days. Germinated embryos were transferred from petri dishes to glass culture tubes.

*Method 3.* Seeds extracted from fruits in laminar flow hoods were placed in sterile empty plastic petri dishes and carefully examined on a mechanism prepared with fluorescent light, and seeds containing embryo were selected and transferred to another sterile empty plastic petri dishes. Embryos were excised from these seeds under stereo binocular microscope, and the seeds were placed into culture tubes containing nutrient medium, as in Method 1.

*Method 4.* Seeds, without exposure to any treatment, were directly sown in sterile plastic petri dishes containing liquid nutrient media (80–120 seeds/petri). As a nutrient medium, again, CP medium with 30 g L^−1^ sucrose, 0.08 mg L^−1^ Vitamin B12, 0.02 mg L^−1^ IAA and E20A medium without agar were used. Sown seeds were observed for 20 days, and when haploid embryos were seen, they were transferred to culture tubes containing nutrient media.

*Method 5.* All the seeds were waited in 15% sodium hypochlorite for 10 minutes for surface sterilization and washed 3–5 times with sterile water. Then, they were sown into glass petri dishes containing CP medium with 30 g L^−1^ sucrose, 8 g L^−1^ agar, 0.08 mg L^−1^ Vitamin B12, 0.02 mg L^−1^ IAA and E20A medium with 8 g L^−1^ agar. Seeds, observed for 10 days, were transferred to nutrient media in glass culture tubes after the haploid embryo detection.

All cultures were incubated in the growing room at 25°C temperature and under 8-hour dark and 16-hour light photoperiod conditions. The experiment was designed in a completely randomized experimental design with three replications and five fruits included per replication. The number of seeds in each fruit and those containing the embryo was detected, the required time for each method was recorded, and unit costs of each method were compared. Total embryo number per fruit, the number of embryos transforming into plant, the number of developed plants, amount of infection, time for opening a fruit, and cost of labor force per embryo were observed to compare the methods. Variance analysis was conducted to evaluate the results, and Tukey test was used for controlling the significance of the differences. 

## 3. Results and Discussion

When the methods were compared, seed numbers per fruit were close to each other, and infection was not observed except in Method 4 during any stage of the study as shown in Table 1. Therefore, methods were compared in terms of embryo number per fruit. Method 1 was found to be most successful with 46 embryos fruit^−1^, followed by Method 3 with 42 embryos fruit^−1^ and Method 2 (CP) with 36 embryos fruit^−1^. The number of obtained embryos was found to be 17 embryos fruit^−1^ in Method 2 (E20A), 7 embryos fruit^−1^ in Method 5 (CP), and 3 embryos fruit^−1^ in Method 5 (E20A). Embryo germination could not be provided in Method 4 because of infection. 

Average embryo number extracted from one fruit in different methods was given in Table 2. There was no statistical difference among Methods 1, 2 (CP), and 3. Accordingly, on average, 3.30 embryos fruit^−1^ were obtained from Method 1, 3.10 embryos fruit^−1^ from Method 3, and 2.40 embryos fruit^−1^ from Method 2 (CP). The embryo number was 1.10 embryos per fruit in Method 2 (E20A); whereas the average embryo number was found to be 0.50 embryos per fruit in Method 5 (CP), it was 0.20 embryos per fruit in Method 5 (E20A). 

To compare the methods in terms of time for obtaining the embryo, by keeping the time the fruit was cut, total time, seed extraction of each method, time for rescuing the embryo, and embryo number per fruit were identified (Table 3). In the present study, only seed extraction from the fruit was calculated as approximately 31.4 minutes for one fruit in each method. In the method of   “inspecting seeds one by one” as the control treatment, roughly 3.3 embryos were obtained from one fruit, and the time for opening seeds in fruits, finding the embryos, and sowing on the media in culture tubes was about 103.5 minutes. 

In Method 2, the rate of rescued embryos per fruit was 2.4, and all the seeds in each fruit were sown in sterile plastic petri dishes containing nutrient media in approximately 56.3 minutes. When compared with Method 1, there was no statistical difference in terms of the obtained embryos number, but this method has an advantage in terms of the time spent per fruit. 

Seeds containing embryo could be easily selected on the light source in Method 3. These seeds were opened embryos were taken and transferred to the nutrient media. Approximately 3.1 embryos were rescued per fruit, and this method took roughly 48.8 minutes. When compared with Method 1, the process was completed in less than half of the time; when evaluated in terms of embryo number per fruit, it was found to be statistically in the same group with Method 2. When compared with the study conducted by Lotfi and Salahi [16], who stated that they examined more than 400 seeds in one hour, it was seen that roughly 1500 seeds were inspected per hour in our study. The same researchers noted that opening fruits one by one took hours. In our study, we conducted with about similar seed number; opening one fruit was completed in 1.5–2 hours. 

 Another negative side of Method 1 is that embryos may become damaged in case it is performed by people with inadequate experience. Moreover, small and immature embryos at the globular stage may not be noticeable unless seen through a binocular microscope. Apart from these methods, in “after-sterilization sowing,” all the seeds in each fruit were sown in petri dishes containing nutrient media in approximately 70.0 minutes. The most important reason of the increase in duration here is the time spent for sterilization of the seeds. In this method, embryo yield was quite low; the cause of which was thought that sterilization fluid passed through the unripe embryo shell and damaged the embryo. 

 Method 4 was unsuccessful because of infection. In a study carried out by Lotfi et al. [14], they remarked that infection occurred in some of the petri dishes, and they lost 30% of the fruits used because of this reason. These researchers examined two different sterilization methods in their study and stated that seed sterilization was more successful than fruit sterilization. In another study conducted by Lotfi and Salahi [16], they indicated that 22% of the seeds transferred to the fluid media got infected. They specified that losses due to infections were 7.8% in seeds disinfected by Clorox, whereas it was 35% in fruit disinfection. Infection is a big problem in fluid culture because infection can quickly spread from one seed to the media in a short time. 

 The cost of the 5 different methods used in obtaining embryos is rather important as well. For this reason, the number of haploid plants obtained through each method and total labor force cost for doing this were examined. With the calculations as basis, the cost of each obtained haploid plants was almost 30$, and 9.49 $/day was paid for the worker extracting seeds from the fruit, and 18.43 $/day was paid for the ones working in embryo rescue from the seeds. In cost account, daily working hour of a worker was calculated as 480 minutes. Because almost all obtained embryos transformed into plant in our study, embryo number was used also as plant number. Unit haploid plant cost was found by dividing calculated total cost by the obtained haploid plant number. By comparing the unit costs calculated for each method, the method with the least cost was determined (Table 4).

 About 85.52 plants are produced, and $2565.6 is earned in Method 3. This method is followed by Method 2 (CP); the number of obtained plant was calculated as 46.26, and the cost was $1387.8. Similar number of plant was obtained as a result of Method 2 (E20A) whose embryo number was lower than Method 1. Furthermore, considering the labor cost, it was apparent that more income was gained. Thus, 21.20 plants and $636.00 were obtained in Method 2 (E20A). In Method 1, the number of the obtained plants was 21.96, and the income was $658.8. In Method 5 (CP), the number of plants was 5.33, and income was $159.9, whereas in Method 5 (E20A), the plant number was 2.13, and the income was $56.61. Considering that about 1000 people can work for a comprehensive improvement practice, when obtained data are calculated according to 1000 plants, Method 3 lasts shortest with 11.6 days; for this reason, the labor cost in this method will be very low, $213.78. Method 2 (CP) lasted 21.7 days, with a total cost of $399.93. Method 2 (E20A) lasted for 46 days with a cost of $847.78. Method 1 lasted for 46.1 days, with a cost of $849.62. Method 5 (CP) lasted 202.0 days and costed $3722.86. Method 5 (E20A) lasted 473.5 days, with a cost of $8726.60. As a consequence, when we examine the labor force cost necessary to obtain 1 haploid plant, it appears that $0.2 for Method 3, $0.4 for Method 2 (CP), $0.8 for Method 2 (E20A), $0.8 for Method 1, $3.7 for Method 5 (CP), and $8.7 for Method 5 (E20A) are required. 

## 4. Conclusion

 When embryo rescue methods were compared, the highest values in terms of embryo numbers per fruit were found in Methods 1, 2, and 3, and there was no statistical difference among these methods. When the methods were compared in terms of duration, Method 1, which used as a control in the experiment, took the longest time, and Method 3 took the shortest time. This method lasted shorter than the other methods because the embryos of the seeds in petri dishes placed on a lighting mechanism seemed more easily. When evaluated in terms of cost, Method 3 provided the highest income, and Method 5 (E20A) brought the least income. As a consequence of the methods that were tried to extract haploid embryos more easily and effectively, many haploid embryos were obtained in a short time through Method 3 and Method 2 (CP). Improvement practices of local species in our country, which has a wide genetic potential in terms of melon, will gather pace, and the dihaploidization method will be used more commonly and effectively in our country's improvement practices. 

## Figures and Tables

**Figure 3:**
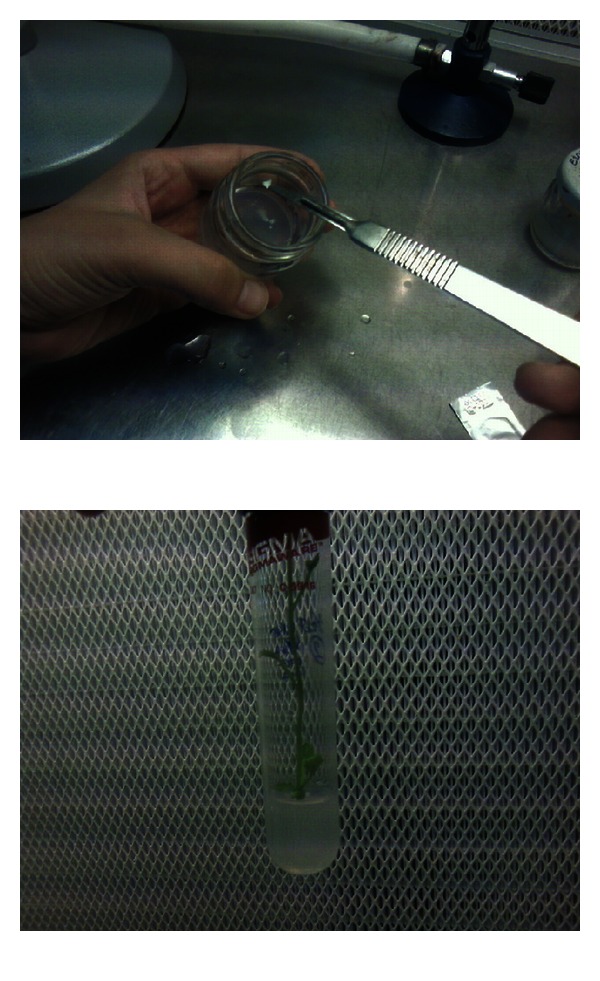
A haploid melon embryo and a plant obtained through germination of this embryo.

**Table 1:** Comparison of seed number per fruit, embryo number, the number of embryos transforming into plant, the number of developed plants, and amount of infection in melon according to methods (Figure 3).

Methods	Seed number per fruit	Embryo number	GEN	GPN	IN
Method 1	1206	46	44	44	—
Method 2 (CP)	1132	36	36	36	—
Method 2 (E20A)	1293	17	17	17	—
Method 4 (CP)	—	—	—	—	—
Method 4 (E20A)	—	—	—	—	—
Method 5 (CP)	1353	7	7	7	—
Method 5 (E20A)	1252	3	3	3	—
Method 3	1249	42	42	42	1

GEN: germinated embryo number.

GPN: growing plant number.

IN: infection number.

**Table 2:** Average embryo number per fruit obtained from different methods.

Methods	Embryos per fruit
Method 1	3.30 a
Method 2 (CP)	2.40 a
Method 2 (E20A)	1.10 b
Method 5 (CP)	0.50 b
Method 5 (E20A)	0.20 b
Method 3	3.10 a
LSD % 5	1.194

**Table 3:** Average embryo number per fruit obtained through different methods and time.

Methods	Embryos/fruit	Time (seed extraction excluded-minutes/fruit)	Total time (minute/fruit)
Method 1	3.30 a	72.1	103.5
Method 2 (CP)	2.40 a	24.9	56.3
Method 2 (E20A)	1.10 b	24.9	56.3
Method 5 (CP)	0.50 b	45.0	70.00
Method 5 (E20A)	0.20 b	45.0	70.00
Method 3	3.10 a	17.4	48.80
Method 4 (CP)	—	5.1	36.5
Method 4 (E20A)	—	5.1	36.5

**Table 4:** Labor force and embryo costs according to time in different embryo rescue methods.

Methods	Embryo number/day	Income ($)	For 1000 plants		
			Time (day)	Labor ($)	Haploid plant/labor force ($)
Method 1	21.96	658.8	46.1	849.62	0.8
Method 2 (CP)	46.26	1387.8	21.7	399.93	0.4
Method 2 (E20A)	21.20	636.00	46.0	847.78	0.8
Method 3	85.52	2565.6	11.6	213.78	0.2
Method 5 (CP)	5.33	159.9	202.0	3722.86	3.7
Method 5 (E20A)	2.13	63.9	473.5	8726.60	8.7

## Abbreviations

Y2 and Y3: Melon genotypes selected from the third backcross (BC3) population of Yuva (local recurrent cultivar)
CP: A nutrient medium developed by Chee et al. [15].
